# Complement Activation in Association with Markers of Neutrophil Extracellular Traps and Acute Myocardial Infarction in Stable Coronary Artery Disease

**DOI:** 10.1155/2020/5080743

**Published:** 2020-03-26

**Authors:** Karsten E. Kluge, Miriam S. Langseth, Trine B. Opstad, Alf Å. Pettersen, Harald Arnesen, Theis Tønnessen, Ingebjørg Seljeflot, Ragnhild Helseth

**Affiliations:** ^1^Center for Clinical Heart Research, Department of Cardiology, Oslo University Hospital Ullevål, Norway; ^2^University of Oslo, Norway; ^3^Department of Internal Medicine, Ringerike Hospital, Vestre Viken, Norway; ^4^Department of Cardiothoracic Surgery, Oslo University Hospital, Norway; ^5^Department of Cardiology, Oslo University Hospital Ullevål, Norway

## Abstract

Complement activation and neutrophil extracellular traps (NETs) have both been suggested to drive atherosclerotic plaque progression. Although experimental studies suggest interplay between these two innate immunity components, the relevance in patients with coronary artery disease (CAD) is unclear. The aim of this study was to assess associations between complement activation and NETs in patients with stable CAD and examine the role of complement activation on clinical outcome. Blood samples from a cohort of patients with angiographically verified stable CAD (*n* = 1001) were analyzed by ELISA for the terminal complement complex (TCC) and by relative quantification for gene expression of the C5a receptor 1 (C5aR1) as markers of complement activation. As markers of NETs, dsDNA was analyzed by fluorescent nucleic acid stain and myeloperoxidase-DNA (MPO-DNA) by ELISA. Clinical outcome was defined as unstable angina, nonhemorrhagic stroke, acute myocardial infarction (MI), or death (*n* = 106, whereof 36 MI). Levels of TCC and C5aR1 were not significantly correlated to dsDNA (TCC: *r* = −0.045, *p* = 0.153; C5aR1: *r* = −0.060, *p* = 0.434) or MPO-DNA (TCC: *r* = 0.026, *p* = 0.414; C5aR1: *r* = 0.123, *p* = 0.107). When dividing TCC and C5aR1 levels into quartiles (Q), levels of MPO-DNA differed significantly across quartiles (TCC: *p* = 0.008, C5aR1: 0.049), while dsDNA did not (TCC: *p* = 0.181, C5aR1: *p* = 0.771). Patients with TCC levels in Q4 had significantly higher levels of MPO-DNA than Q1-3 (*p* = 0.019), and C5aR1 levels in Q3-4 had significantly higher levels of MPO-DNA than Q1-2 (*p* = 0.046). TCC levels did not differ between patients experiencing a clinical endpoint or not, but high levels were associated with increased risk of acute MI (OR. 1.97, 95% CI: 0.99-3.90, *p* = 0.053) during two-year follow up, also when adjusted for relevant covariates. In conclusion, TCC and C5aR1 were moderately associated with the NET marker MPO-DNA, and TCC levels were related to the risk of future MI in this cohort of patients with stable CAD.

## 1. Introduction

The primary cause of coronary artery disease (CAD) is atherosclerosis, a slowly progressing plaque-building inflammatory process of the coronary arteries with subsequent risk of plaque erosion or rupture, thrombosis, and myocardial infarction (MI) [1, 2]. The clinical significance of immune activation during atherogenesis and atherothrombosis has recently become obvious [3, 4], underpinned by the CANTOS trial, showing that the administration of anti-interleukin- (IL-) 1*β* to patients with established CAD resulted in a lower risk of recurrent cardiovascular events compared to placebo [5].

The complement system is an essential part of the innate immune system, consisting of more than 40 soluble and cell-bound proteins of which many circulate as inactive precursor proteins in the steady state [6, 7]. Upon activation of one or more of the three activating pathways—the classical pathway (CP), the lectin pathway (LP), and the alternative pathway (AP)—consecutive cleavage of complement proteins eventually leads to the formation of a terminal C5b-9 complement complex (TCC) which, when membrane bound, creates a hole in the cellular membrane leading to cell lysis [8, 9]. Although previously viewed as mainly having antimicrobial functions, the complement system is now considered an integral player in atherosclerosis progression. Complement seems to induce proinflammatory effects in endothelial cells, proliferation and proinflammatory signaling in smooth muscle cells, and promoting inflammation through the anaphylatoxins C3a and C5a, which can bind to their receptors, C3aR and C5aR within the plaque and on leukocytes [10–19]. Higher circulating levels of complement proteins like C3, C3a, C4, and C5a have been associated with the increased risk of cardiovascular events [20]. In acute cardiovascular events, the complement system is suggested to contribute both to thrombosis [21, 22] and to the ischemia-reperfusion (IR) injury following restoration of blood flow to the ischemic tissue [23–25].

Also, part of the innate immune system are neutrophil extracellular traps (NETs), fragments of nuclear material studded with granule proteins released from neutrophils upon activation [26]. NETs were initially considered to exert mainly antimicrobial functions [27, 28] but have later been recognized in the pathophysiology of many other diseases, including CAD [29]. NETs have been proposed as mediators of endothelial dysfunction, as well as elicitors of inflammatory responses from plasmacytoid dendritic cells and macrophages, all driving atherosclerotic progression [30]. In patients with stable CAD, levels of circulating NET markers have been associated with CAD severity and clinical outcome [31], and in acute myocardial infarction (MI), circulating NET markers as well as the amount of NETs in aspirated coronary thrombi have been associated with myocardial infarct size [32, 33].

Although experimental data have indicated a significant interplay between complement activation and NETs by being potent activators of each other, thus, propagating an inflammatory reaction [34], this has not yet been explored in clinical studies. We recently showed that markers of NETs were associated with adverse clinical outcome in patients with stable CAD [35]. Based on the hypothesis of a positive feedback loop between complement activation and NET release, we now aimed to investigate whether complement activation by levels of TCC and expression of C5aR1 was associated with levels of the NET markers dsDNA and myeloperoxidase-DNA (MPO-DNA) in patients with stable CAD. Any relation between complement activation, systemic inflammation, and adverse clinical outcome was further assessed.

## 2. Materials and Methods

### 2.1. Study Population

A cohort of patients (*n* = 1001) with angiographically verified CAD were included in the Aspirin nonresponsiveness and Clinical Endpoint Trial (ASCET) at Oslo University Hospital, Ullevål, during 2004-2008, described in details elsewhere [36]. After at least one week on aspirin monotherapy, patients were randomly assigned to either continue aspirin or switch to clopidogrel. All patients were followed for two years for clinical endpoints. Patients with an indication for additional anticoagulant therapy were excluded, as were pregnant and breast-feeding women and patients with alcoholism, psychiatric disease, or other conditions that could reduce compliance. All participants gave written informed consent. The study is in accordance with the declaration of Helsinki, was approved by the regional ethics committee, and is registered at http://www.clinicaltrials.gov (NCT00222261).

Diabetes was defined as previously diagnosed type 1 diabetes mellitus (T1DM), type 2 diabetes mellitus (T2DM), or fasting glucose levels > 7 mmol/L. Hypertension (HT) was defined as previously diagnosed HT, and smoking was defined as current smoking or cessation less than three months prior to study inclusion.

### 2.2. Laboratory Methods

Venous blood samples were collected in a fasting state before morning medication (8:00-10:30 am). EDTA plasma was stored on ice and separated within 30 min by centrifugation at 4°C at 3000 *g* for 20 min. The samples were then stored at -80°C until analyzed.

Plasma levels of TCC were quantified using a commercially available immunoassay (human TCC, Hycult Biotech, Uden, The Netherlands); results are presented as arbitrary units (AU). The interassay variation coefficient (CV) was 8%. PAXGene Blood RNA tubes were collected for RNA extraction from circulating leukocytes in a randomly selected subset of 173 patients and analyzed for gene expression of the complement receptor C5aR1. Total RNA was extracted using the PAXGene Blood RNA kit (Pre-Analytix) with an extra cleaning step (RNeasy MinElute Cleanup kit, Qiagen). RNA purity and quantity was tested on the NanoDrop, ND-1000 (Saveen Werner, Sweden). An equal amount of total RNA per experiment (100 ng) was reversely transcribed into complementary DNA (cDNA) by the use of qScript cDNA SuperMix (Quanta Biosciences Inc., Gaithersburg, USA). Leukocyte expression of C5aR1 (Hs00704891_s1) was normalized to *β*-2-microglobulin (Hs99999907_m1). The gene expression analyses were performed on the VIIa7 instrument (Applied Biosystems Life Technologies, Foster City, CA, USA) using TaqMan Universal PCR Master Mix, NoAmpErase UNG (Applied Biosystems), as relative quantification (RQ) (2^-*ΔΔ*Ct^ method) (Livak and Schmittgen, 2001).

Markers of NETs, MPO-DNA and dsDNA, were analyzed as previously described [35]. In brief, MPO-DNA complexes were quantified in a serum using an enzyme-linked immunosorbent assay (ELISA) technique where anti-MPO monoclonal antibodies (AbD Serotec, Hercules, CA, USA) were coated to plates and incubated overnight at 4°C. After blocking, patient serum and peroxidase-labeled anti-DNA monoclonal antibody (Cell death detection kit; Roch Diagnostics, Mannheim, Germany) were added. After incubation and washing, a peroxidase substrate was added and absorbance measured after 40 min. Values were reported as optical density (OD) units. Serum dsDNA levels were quantified using a fluorescent nucleic acid stain, quant-iT PicoGreen (Invitrogen Ltd., Paisley, UK), and a Fluorskan Ascent fluorometer (Thermo Fisher Scientific Oy, Vantaa, Finland). The interassay CVs for the analyses were 10.5% for MPO-DNA and 7% for dsDNA [35].

### 2.3. Clinical Endpoints

The primary clinical endpoint was defined as a composite of unstable angina, acute MI, nonhemorrhagic stroke, or death from any cause, whichever occurred first. A committee without access to laboratory data evaluated the endpoints.

### 2.4. Statistical Analyses

Data is presented as mean ± SD, median (25th and 75th percentile), or numbers (%) as appropriate. The unpaired Student *t*-test, Mann–Whitney *U* test, and Kruskal-Wallis test were used to determine differences between groups as appropriate. Proportional data was compared using the chi-squared test. Correlation analyses were performed using Spearman's rho. Data was further divided into quartiles to assess any nonlinear relationships and potential thresholds. Significance of trends was assessed using the Mantel-Haenszel test for linear-by-linear association. Univariate logistic regression was used to assess the impact of TCC on the risk of experiencing a composite clinical endpoint or an acute MI. Potential covariates were adjusted for in a multivariate logistic regression analysis. *p* values of ≤ 0.05 were considered statistically significant, and all statistical analyses were performed using IBM SPSS statistics v.25.

## 3. Results

### 3.1. Study Population

Baseline characteristics of the total population and according to having a clinical endpoint or not are shown in Table 1. The number of composite endpoints recorded after two years was 106. In the total population, 96.8% were Caucasian, 21.8% were women, and the mean age was 62 years. One-fifth were smokers, and one-fifth had diabetes. Nearly all study participants used statins, and about 75% used beta blockers. Almost half of the study participants (*n* = 436) had previously suffered an acute MI, while 2.7% had suffered ischemic stroke. The two latter proportions were significantly higher among those who experienced an endpoint. Clinical outcome did not differ between the aspirin and clopidogrel group, and the rest of the article will not address differences between them.

### 3.2. Associations between Complement Markers and NETs

Neither TCC levels nor gene expression levels of C5aR1 were significantly correlated with dsDNA or MPO-DNA levels. Nor did the two complement markers intercorrelate significantly (Table 2).

**Figure 1:**
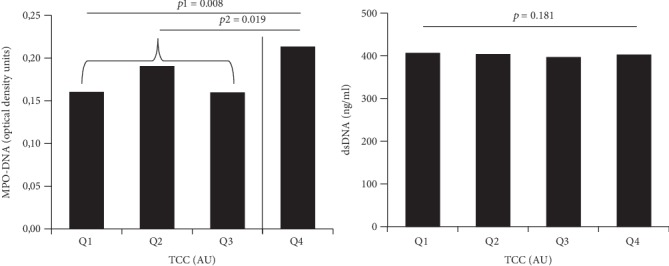
TCC levels in quartiles related to levels of NET markers. Quartiles (*Q*) of TCC related to markers of NETs. Solid line indicates the threshold for dichotomizing levels. *p* and *p*1 refers to the Kruskal-Wallis test across quartiles; *p*2 refers to the Mann–Whitney *U* test. TCC: terminal complement complex; AU: arbitrary units.

When dividing TCC levels into quartiles, MPO-DNA differed significantly across quartiles (*p* = 0.008), whereas dsDNA did not (*p* = 0.181) (Figure 1, Kruskal-Wallis test). Based on the quartile distribution and a potential threshold between the highest quartile (Q4) and the lower three quartiles (Q1-3) of TCC, data was dichotomized at the third quartile. As outlined in Figure 1, MPO-DNA levels were significantly higher in patients with TCC levels in Q4 vs. Q1-3 (*p* = 0.019).

**Figure 2:**
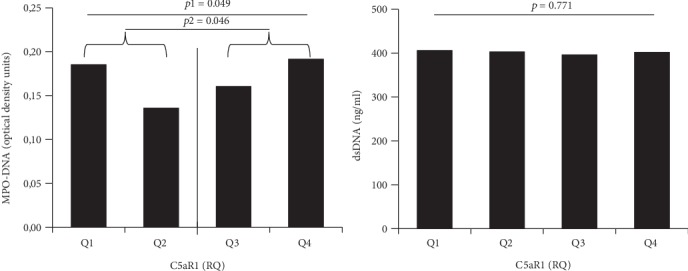
C5aR1 gene expression in quartiles related to levels of NET markers. Quartiles (*Q*) of C5aR1 expression as related to markers of NETs. Data was dichotomized at median level (solid line). *p* and *p*1 refers to the Kruskal-Wallis test across quartiles; *p*2 refers to the Mann–Whitney *U* test. RQ: relative quantification.

When dividing C5aR1 gene expression levels (*n* = 173) into quartiles, MPO-DNA likewise differed significantly across quartiles (*p* = 0.049), while dsDNA did not (*p* = 0.771) (Figure 2, Kruskal-Wallis test). Based on the quartile distribution, C5aR1 levels were further dichotomized into levels below and above median levels, and as outlined in Figure 2, MPO-DNA levels were significantly higher in patients with above-median vs. below-median C5aR1 gene expression (*p* = 0.046).

### 3.3. Associations between C-Reactive Protein and Markers of Complement Activation and NETs

As shown in Table 3, concentrations of C-reactive protein (CRP) were significantly correlated to concentrations of TCC and dsDNA, but not to MPO-DNA or C5aR1 expression.

### 3.4. TCC and Traditional Cardiovascular Risk Factors

As shown in Table 4, levels of TCC were significantly elevated in patients older than the median age of 62 years, women, and in patients without a previous acute MI (Mann–Whitney *U* test). TCC levels did not differ significantly with regard to smoking status, the presence of HT, or diabetes (Mann–Whitney *U* test). Concentrations of TCC were weakly, yet statistically significantly, associated with neutrophil counts (*r* = 0.102, *p* = 0.001, Spearman's rho).

### 3.5. TCC and Clinical Endpoints

**Figure 3:**
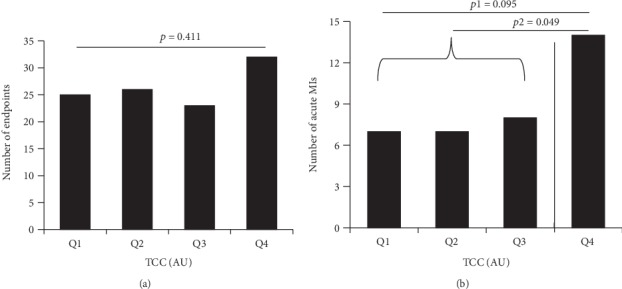
TCC in quartiles related to a number of clinical endpoints and a number of acute MIs. *p* value and *p*1 value refer to the Mantel-Haenszel test for linear-by-linear associations. Solid line indicates the threshold for dichotomizing levels, the *p*2 value refers to the chi-square test. MI: myocardial infarction; TCC: terminal complement complex; AU: arbitrary units.

There was no significant difference in concentrations of TCC between the group that did (*n* = 106) and did not reach a clinical endpoint (2.985 AU vs. 2.975 AU, *p* = 0.800, Mann–Whitney *U* test), nor when dividing TCC into quartiles (*p* = 0.411, Figure 3(a), Mantel-Haenszel test). When analyzing the group that experienced an acute MI (*n* = 36), however, patients with TCC levels in Q4 were significantly more likely to experience a MI than patients with TCC levels in Q1-3 (*p* = 0.049, Figure 3(b), chi-squared test).

Univariate logistic regression analysis showed an elevated risk of MI for patients with TCC levels in the highest quartile with an odds ratio (OR) of 1.97 (95% confidence interval (CI): 0.99-3.90, *p* = 0.053). The enhanced risk remained evident when controlling for age, sex, CRP, current smoking, and previous acute MI in a multivariate regression analysis (OR = 2.14, CI: 1.05-4.36, *p* = 0.037) (Table 5).

## 4. Discussion

Despite the observational studies in humans and experimental studies in animals showing that the complement system exerts detrimental effects on atherosclerosis [8], limited data exists regarding its role in the progression of stable CAD in the clinical setting. To our knowledge, this is the largest study investigating complement activation in patients with stable CAD and the first to relate complement to circulating NET markers in such a population. We have recently shown the impact of NETs in this population [35], and due to the increasing evidence of reciprocal activation between complement activation and NETs [34], we hypothesized that they would activate each other, thus, propagating their individual negative effects on the progression of the disease [8, 30, 37].

The observed association between MPO-DNA and the TCC suggests that complement activation and NETs indeed could be interrelated. The activation of either complement or NETs could, through several mechanisms [34], activate the other system—causing a self-amplifying cycle leading to significant activation of both systems. In line with this, C5aR1 has been shown to prime neutrophils for NETs release [38] and also to activate NETs release directly when neutrophils are pretreated with interferon-*γ* (IFN-*γ*) [39]. Our observation that patients with above median C5aR1 expression had significantly higher levels of MPO-DNA compared to those with below median expression supports an association between complement regulation and NETs and is in line with the abovementioned observations for MPO-DNA and TCC. The lack of association between TCC, C5aR1, and dsDNA is worth noticing but could be due to the proposed low specificity of dsDNA as a marker of NETs [40].

TCC levels were significantly higher with older age, as also previously reported in healthy subjects [41]. However, in contrast to our observations, complement activity was found to be higher in men than in women in this previous cohort [41]. Our observation of higher TCC levels in women could be attributed to the fact that women in our study were significantly older than the men (*p* < 0.001). The significantly lower levels of TCC in patients with previous acute MI might be due to the use of medication as secondary prophylaxis in our cohort. It may also be possible that the myocardium, after being damaged by ischemia, loses some of its ability to inhibit assembly and binding of TCC, most likely due to decreased expression of the complement inhibitor CD59 [42, 43]. Membrane-bound TCC has been shown to be present in the myocardium up to three years after an infarction [44], indicating a long-term depression of ability to inhibit complement activation, and thus, an increased susceptibility to complement-mediated damage. Thus, the significantly lower concentration might reflect an increased proportion of cell- (cardiomyocyte-) bound TCC in patients with a history of MI.

Circulating TCC levels were not associated with the primary composite endpoint after two years in our population—despite some reports of a predictive value of the TCC in acute MI [45, 46]. Patients in the highest quartile (*Q*4) of TCC, however, had a higher risk of acute MI and regression analysis showed that patients with highest TCC levels had increased risk of acute MI during the follow-up time, also when adjusting for age, gender, previous acute MI, smoking status, and CRP. Even though TCC and CRP correlated significantly, patients with high TCC had increased risk of future acute MI also after adjusting for CRP; this could indicate that TCC represent more than traditional inflammation, alternatively a separate inflammatory pathway. The observed association between TCC and future acute MI is in line with reports of complement proteins like C3, C5, C3a, and C5a as predictors of future cardiovascular events in both healthy populations and populations with peripheral artery disease [20], as well as reports of the complement system exerting deleterious effects on atherosclerosis progression [8].

### 4.1. Limitations

Even though TCC is a well-established marker of complement activation, it does not necessarily reflect the amount of cell-bound complement proteins, a balance that is influenced by individual factors such as levels of complement inhibitors [47]. Considering that complement [48, 49] and C5aR1 [17, 18] activation have both been reported within atherosclerotic plaques, general complement activation might not be an accurate reflection of complement activity within the atherosclerotic lesion. It may be suggested that *intralesional* complement activity is the most influential on atherosclerosis progression; thus, measuring circulating TCC does not give us the complete picture. In addition, the methods used for measuring NETs pose a well-known challenge [40]. As previously discussed [35], dsDNA could in principle be released from any nucleated cell, and the ELISA for measuring MPO-DNA is in need of standardization. Nevertheless, MPO-DNA is at present considered the most specific circulating NET marker [40]. Lastly, all included patients were treated with aspirin for at least a week before inclusion and nearly all used statins. Aspirin has been shown to inhibit complement activation and NET formation [50–52], and statin use has been shown to both stimulate and inhibit NET formation [53–56]; thus, this medication may have influenced the observed associations in our cohort.

## 5. Conclusions

In this cohort of patients with stable CAD, high circulating levels of TCC and expression of C5aR1 are associated with the NET marker MPO-DNA, suggesting a clinically relevant interplay between complement activation and NET release. TCC levels did not associate with the composite clinical endpoint, but with the risk of acute MI during two years follow-up. Whether circulating complement protein levels represent an adequate reflection of complement's local effect on atherosclerotic coronary plaque progression remains to be explored.

## Figures and Tables

**Table 1:** Baseline characteristics of the total study population and according to clinical endpoints.

	Total study population (*n* = 1001)	Endpoint (+, *n* = 106)	Endpoint (÷, *n* = 895)	*p* value
Age, mean (range)	62 (36-81)	63 (41-80)	62 (36-81)	0.385
Female gender	218 (21.8)	23 (21.7)	195 (21.8)	0.983
Smoking	203 (20.3)	23 (21.7)	180 (20.1)	0.666
Hypertension	556 (55.5)	63 (59.4)	493 (55.1)	0.401
Diabetes	201 (20.1)	24 (22.6)	177 (19.8)	0.493
BMI (kg/m^2^)	27.1 (24.6, 29.6)	27.4 (24.4, 29.9)	27.2 (24.9, 29.5)	0.717
Previous acute MI	436 (43.6)	57 (53.8)	379 (42.3)	0.026
Previous ischemic stroke	27 (2.7)	6 (5.7)	21 (2.3)	0.047
Total leukocyte count (×10^9^/L)	6.2 (5.3, 7.4)	6.5 (5.6, 8.0)	6.2 (5.3, 7.4)	0.118
Neutrophil count (×10^9^/L)	3.4 (2.7, 4.2)	3.5 (2.8, 4.5)	3.4 (2.7, 4.2)	0.075
Platelet count (×10^9^/L)	228 (195, 264)	224 (190, 270)	228 (195, 264)	0.965
Total cholesterol (mmol/L)	4.53 ± 0.95	4.53 ± 0.99	4.55 ± 0.98	0.910
LDL-cholesterol (mmol/L)	2.53 ± 0.83	2.51 ± 0.75	2.53 ± 0.84	0.852
HDL-cholesterol (mmol/L)	1.34 ± 0.41	1.33 ± 0.39	1.34 ± 0.41	0.988
Triglycerides (mmol/L)	1.29 (0.93, 1.82)	1.5 ± 0.77	1.48 ± 0.76	0.866
Fasting glucose (mmol/L)	5.5 (5.0, 6.2)	5.6 (5.0, 6.5)	5.5 (5.0, 6.2)	0.521
C-reactive protein (mg/L)	2.23 (1.03, 4.11)	2.46 (1.24, 4.81)	2.22 (0.99, 3.97)	0.100
Medication				
Statins	983 (98.3)	105 (99.1)	878 (98.1)	0.524
Beta blockers	756 (75.8)	78 (73.6)	678 (75.8)	0.682
ACE inhibitors	263 (26.4)	32 (30.2)	231 (25.8)	0.320

Values are given as mean (±SD), median (25th and 75th percentiles), or numbers (%) as appropriate. *p* values refer to differences between the group with endpoint or not. Mann–Whitney *U* test, Student's *t*-test, or chi-squared test as appropriate. BMI: body mass index; MI: myocardial infarction; LDL: low-density lipoprotein; HDL: high-density lipoprotein.

**Table 2:** Coefficients of correlations between complement and NET markers (Spearman's rho).

C5aR1	dsDNA	MPO-DNA
TCC		
*r* = −0.116	*r* = −0.045	*r* = 0.026
*p* = 0.132	*p* = 0.153	*p* = 0.414
C5aR1		
—	*r* = −0.060	*r* = 0.123
—	*p* = 0.434	*p* = 0.107

TCC: terminal complement complex.

**Table 3:** Coefficients of correlations between CRP, complement and NET markers (Spearman's rho).

TCC	C5aR1	dsDNA	MPO-DNA
CRP			
*r* = 0.171	*r* = 0.006	*r* = 0.282	*r* = 0.020
*p* < 0.001	*p* = 0.936	*p* < 0.001	*p* = 0.522

CRP: C-reactive protein; TCC: terminal complement complex.

**Table 4:** TCC levels according to traditional cardiovascular risk factors.

	TCC (AU)	*p* value
*Age (years)*		
≥62	3.006	0.003
<62	2.949	
*Sex*		
M	2.933	0.033
F	3.130	
*Previous acute MI*		
+	2.917	0.016
—	3.023	
*Smoking*		
+	3.027	0.668
—	2.964	
*Hypertension*		
+	3.024	0.210
—	2.918	
*BMI (kg/m^2^)*		
≥30	2.920	0.190
<30	2.992	
*Diabetes*		
+	2.874	0.054
—	3.002	
*LDL (mmol/L)*		
≥1,8	3.007	0.108
<1,8	2.859	

Cut-off levels defined as age at median level (62 years), LDL-C concentration of 1.8 mmol/L (the treatment goal on statin therapy) and BMI of 30 kg/m2 (the WHO definition of obesity). *p* values refer to the Mann–Whitney *U* test. TCC: terminal complement complex; AU: arbitrary units; MI: myocardial infarction; BMI: body mass index; LDL: low-density lipoprotein.

**Table 5:** Determinants of suffering an acute MI.

	OR	95% CI	*p* value
TCC Q4 vs. Q1-3	2.137	1.049, 4.355	0.037
Age	0.994	0.956, 1.033	0.753
Sex (male)	0.766	0.343, 1.712	0.766
CRP	0.982	0.902, 1.068	0.665
Current smoking	1.906	0.892, 4.073	0.096
Previous acute MI	2.242	1.101, 4.569	0.026

*p* value refers to the Wald test. OR: odds ratio; CI: confidence interval; MI: myocardial infarction; TCC: terminal complement complex; CRP: C-reactive protein.

## Data Availability

The dataset used during the current study is not available publicly due to Norwegian legislation about general data protection regulations, but are available from the corresponding author on request.
